# AMHF‐TP: Multifunctional therapeutic peptides prediction based on multi‐granularity hierarchical features

**DOI:** 10.1002/qub2.73

**Published:** 2024-11-27

**Authors:** Shouheng Tuo, YanLing Zhu, Jiangkun Lin, Jiewei Jiang

**Affiliations:** ^1^ School of Computer Science and Technology Xi’an University of Posts and Telecommunications Xi’an China; ^2^ Shaanxi Key Laboratory of Network Data Analysis and Intelligent Processing Xi’an China; ^3^ Xi’an Key Laboratory of Big Data and Intelligent Computing Xi’an China; ^4^ School of Electronic Engineering Xi’an University of Posts and Telecommunications Xi’an China

**Keywords:** deep learning, hypergraph, multifunctional therapeutic peptides, multi‐granularity hierarchical features

## Abstract

Multifunctional therapeutic peptides (MFTP) hold immense potential in diverse therapeutic contexts, yet their prediction and identification remain challenging due to the limitations of traditional methodologies, such as extensive training durations, limited sample sizes, and inadequate generalization capabilities. To address these issues, we present AMHF‐TP, an advanced method for MFTP recognition that utilizes attention mechanisms and multi‐granularity hierarchical features to enhance performance. The AMHF‐TP is composed of four key components: a migration learning module that leverages pretrained models to extract atomic compositional features of MFTP sequences; a convolutional neural network and self‐attention module that refine feature extraction from amino acid sequences and their secondary structures; a hypergraph module that constructs a hypergraph for complex similarity representation between MFTP sequences; and a hierarchical feature extraction module that integrates multimodal peptide sequence features. Compared with leading methods, the proposed AMHF‐TP demonstrates superior precision, accuracy, and coverage, underscoring its effectiveness and robustness in MFTP recognition. The comparative analysis of separate hierarchical models and the combined model, as well as with five contemporary models, reveals AMHF‐TP’s exceptional performance and stability in recognition tasks.

## 1 INTRODUCTION

In the field of biomedicine, peptides [1] play a crucial role in cell signaling, drug development, and research. Multifunctional therapeutic peptides (MFTP) are especially significant due to their various biological activities, including anti‐angiogenic, antimicrobial, anti‐cancer, anti‐diabetic, and anti‐inflammatory properties, making them a research and application hotspot [2, 3]. Predicting the biological activities and roles of MFTP is vital for understanding their mechanisms and impact on human health.

However, traditional methods for predicting MFTP activity rely on time‐consuming and expensive laboratory experiments. Reducing cost and increasing speed are major technical challenges in MFTP research. The significant reduction in sequencing costs and the rapid advancement of machine learning and deep learning technologies provide substantial data and technical support for MFTP research [4, 5]. These approaches use large‐scale biological data, peptide sequence information, and biological features to train neural network models to predict MFTP biological activities, affording new possibilities for peptide design and application.

The application of deep learning in identifying MFTP presents several challenges. Firstly, the limited availability of MFTP data, coupled with an imbalance in sample distribution, can lead to overfitting and diminished model accuracy on novel data. This imbalance tends to favor classes with more samples, which may compromise the model’s performance on less represented classes. Secondly, the inherent biological diversity of MFTP, characterized by their varied structures, sequences, and functions, adds to the complexity of modeling, necessitating advanced algorithms capable of capturing and generalizing the intricate features of different MFTP types. Thirdly, the multifactorial nature of MFTP’s biological activity, influenced by peptide sequences, structures, and biological interactions, requires the development of sophisticated feature representation techniques to effectively harness deep learning. Lastly, the interpretability of deep learning models is a significant concern.

To tackle these challenges, researchers have explored different methods, including increasing the amount of data, utilizing data augmentation techniques, enhancing model architecture, integrating attention mechanisms to emphasize crucial features, and utilizing interpretive models to clarify the decision‐making process of deep learning models.

Previous studies have predominantly utilized conventional machine learning and ensemble learning techniques. For instance, Xiao et al. [6] created a multi‐label classifier called iAMP‐2L, which is based on pseudo amino acid composition and fuzzy K‐nearest neighbor algorithm. This classifier operates on two levels: the first level determines whether a peptide is antimicrobial and the second level identifies its functional types. The aim of this work was to provide fundamental tools and information for basic research and drug development. To tackle data imbalance, Lin et al. [7] proposed a new oversampling technique for synthesizing minority class samples, called ML‐SMOTE, which improves predictive models for functional peptides. Wei et al. [8] developed PEPred‐Suite, a method that uses an adaptive feature representation strategy to learn the most representative features of different peptide types. This approach entails training multiple random forest models for eight different functional peptide types. In their work, Bin et al. [9] developed PredNeuroP, a neuropeptide identification model that uses an ensemble of 45 base learning models, achieving high accuracy. Dai et al. [10] developed a computational method called BBPpred, which effectively identifies blood–brain barrier peptides through logistic regression and feature learning.

In recent years, there has been an increasing interest in identification methods based on deep learning. Grønning et al. [11] developed MultiPep, a tree‐like feature extraction model that uses convolutional neural networks (CNNs) to identify 20 functions of bioactive peptides. They also introduced a new loss function that outperforms the class‐weighted binary cross entropy. Xiao et al. [12] developed a two‐tiered AMP prediction method that combines CNN, bidirectional long short‐term memory (Bi‐LSTM), and support vector machines (SVM) classifiers. This method significantly improves predictive performance in systems containing both single and multifunctional AMPs. Chen et al. [13] presented NeuroPred‐CLQ, a deep learning model based on temporal convolution networks and multi‐head attention mechanisms. This model efficiently identifies neuropeptides and converts their internal relationships into numerical features. Chu et al. [14] introduced TransPHLA, a transformer‐based model for predicting peptide affinity with human leukocyte antigen alleles. They also introduced the automatically optimized mutated peptides program for generating potential peptide vaccines. Li et al. [15] proposed MPMABP, a method that uses CNN and Bi‐LSTM to identify multiple functions of AMPs. They utilized stacked CNNs of five different scales and residual networks. Otovic et al. [16] proposed a hybrid approach for predicting the antimicrobial and antiviral activities of peptides. The approach involves extracting sequence arrangement, physicochemical, topological, and geometrical properties of peptides. Tang et al. [17] developed MLBP, which uses peptide sequence vectors as input and learns continuous feature vectors through CNN and bidirectional gated recurrent units to identify therapeutic actions. Yan et al. [18] proposed PrMFTP, which utilizes a multi‐scale CNN, Bi‐LSTM, and multi‐head self‐attention (MHSA) to extract and learn peptide sequence features. They also designed a class weight optimization scheme to address label imbalance. In addition, Fan et al. [19] introduced ETFC for predicting 21 therapeutic peptides, using an imbalance learning strategy.

Although the methods previously mentioned have yielded significant outcomes, there are still challenges that need addressing. Firstly, conventional machine learning techniques, such as SVM and random forest, exhibit limited proficiency in identifying multifunctional peptides, leading to less‐than‐optimal accuracy. Secondly, there is a deficiency in the investigation of atomic‐level characteristics that are smaller than sequences. Thirdly, the majority of current methods are developed from the ground up, not taking advantage of preexisting models that encapsulate prior knowledge, which results in increased demands on training time and computational resources. Fourthly, while self‐attention mechanisms are beneficial for processing sequence data, they struggle with recognizing local dependencies and are burdened by high computational demands. Lastly, graph neural networks have been utilized for discerning peptide sequence interactions, but they predominantly function on simplistic graphs and only acknowledge binary relationships between nodes, which do not suffice for the representation of more intricate interactions.

**FIGURE 1:**
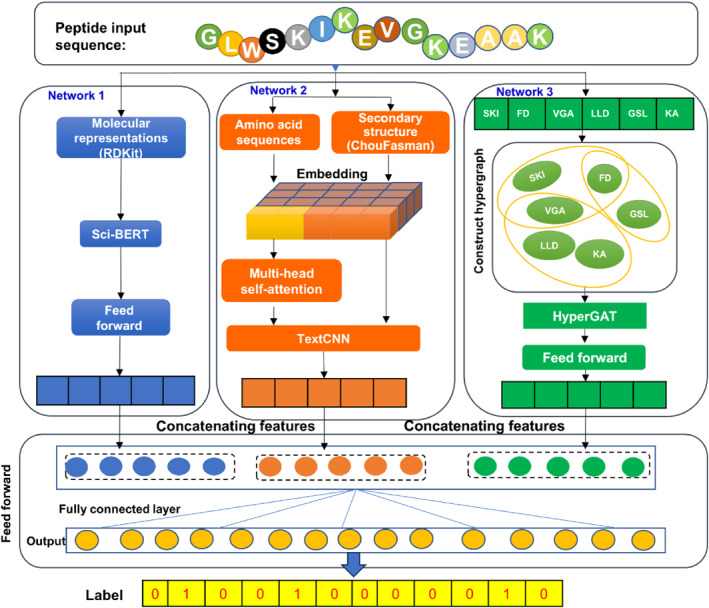
The framework of the AMHF‐TP for MFTP prediction. Network 1 aims to extract features of the amino acid composition of MFTP sequences through transfer learning. Network 2 aims to extract features of amino acid sequences and their secondary structures using convolutional neural networks and self‐attention mechanisms, obtaining features of amino acid sequences. Network 3 aims to capture the high‐order similarity of sequences by constructing a hypergraph. After the extraction through the above three layers of networks, features of atomic sequences, amino acid sequences, and multi‐sequence relationships are obtained, respectively. Finally, it is necessary to pass through a feature fusion layer for the fusion extraction of the three layers of features, thereby obtaining the classification of multifunctional peptides. MFTP, multifunctional therapeutic peptides.

To enhance the prediction ability of MFTP, this study proposes AMHF‐TP, a method for recognizing MFTP that utilize attention mechanisms and multi‐granularity hierarchical features (see Figure 1). This method integrates biological features, sequence information, and interaction networks to improve the accurate prediction of multifunctional peptides. The main contributions of this study are as follows:

1. This study employs transfer learning to extract amino acid atomic composition features of multifunctional peptides, leveraging previously acquired knowledge to identify potential relationships in peptide sequences.
2. Additionally, the study combines CNNs with self‐attention mechanisms to enhance local feature extraction and diversify feature representation from amino acid sequences and their secondary structures.
3. This study builds hypergraphs to extract relational features between peptide sequences and adds complex similarity features.
4. It also extracts peptide sequence features on three levels: atomic, amino acid sequence, and multi‐sequence relation. The study constructs a multi‐granularity hierarchical feature extraction algorithm for multifunctional peptides, fully capturing their potential features.

## 2 RESULTS

This section presents the evaluation of the proposed AMHF‐TP model and its three‐tier feature extraction models. The performance of the AMHF‐TP model is compared with several state‐of‐the‐art models using different metrics.

### 2.1 Dataset construction

This study utilized the MFTP dataset [18], a multi‐label benchmark for functional peptide classification. The dataset comprises 9841 data entries, categorized into 21 different types of peptides, such as antiangiogenic peptide (AAP), anti‐HIV peptide (AHIVP), surface binding peptide (SBP), antifungal peptide (AFP), antibacterial peptide (ABP), antihypertensive peptide (AHP), antiviral peptide (AVP), anticancer peptide (ACP), anti‐inflammatory peptide (AIP), antiparasitic peptide (APP), antiendotoxin peptide (AEP), biofilm inhibitory peptide (BIP), blood–brain barrier peptide (BBP), anticoronavirus peptide (ACVP), anti‐MRSA peptide (AMRSAP), and cell‐penetrating peptide (CPP). The sample categories include dipeptidyl peptidase IV peptide (DPPIP), antidiabetic peptide (ADP), antitubercular peptide (ATP), tumor homing peptide (THP), and quorum‐sensing peptide (QSP). The text describes the distribution of sample categories in Table 1.

**TABLE 1:** Number of samples in the dataset.

Number (*n*)	Peptide
*n* ≤ 500	BBP, BIP, CPP, DPPIP, QSP, SBP, AAP ACVP, AEP, AHIVP, AMRSAP, APP, ATP
500 < *n* ≤ 1000	AVP, THP, ADP, AHP
*n* > 1000	ABP, ACP, AFP, AIP

The dataset was divided into a training set (80%) and a test set (20%). The training set was used for building and tuning the models and hyperparameter optimization, while the test set was used for evaluating their performance.

### 2.2 Evaluation metrics

To assess how well AMHF‐TP performs on multi‐classification problems, we use five common metrics that are suitable for this task: precision, accuracy, coverage, absolute true, and absolute false. These metrics measure different aspects of the quality of the predictions made by AMHF‐TP. The formulas for computing these metrics are given below:

(1) $$\left\{\begin{array}{c} \text{Precision}=\frac{1}{N}\sum_{i=1}^{N}\frac{\left\|y_{i}\cap {\bar{y}}_{i}\right\|}{\left\|{\bar{y}}_{i}\right\|}\\ \text{Accuracy}=\frac{1}{N}\sum_{i=1}^{N}\frac{\left\|y_{i}\cap {\bar{y}}_{i}\right\|}{\left\|y_{i}\cup {\bar{y}}_{i}\right\|}\\ \text{Coverage}=\frac{1}{N}\sum_{i=1}^{N}\frac{\left\|y_{i}\cap {\bar{y}}_{i}\right\|}{\left\|y_{i}\right\|}\\ \text{Absolute}\;\text{true}=\frac{1}{N}\sum_{i=1}^{N}∆\left(y_{i},{\bar{y}}_{i}\right)\\ \text{Absolute}\;\text{false}=\sum_{i=1}^{N}\frac{\left\|y_{i}\cup {\bar{y}}_{i}\left\|-\right\|y_{i}\cap {\bar{y}}_{i}\right\|}{M} \end{array}\right.$$

where *N* is the total number of peptide sequences in the dataset, *M* is the total number of label types in the label set, *y*_*i*_ represents the true label set of the *i*th sample, and ${\bar{y}}_{i}$ represents the predicted label set of the *i*th sample with $∆\left(y_{i},{\bar{y}}_{i}\right)$ defined as follows:

(2) $$∆\left(y_{i},{\bar{y}}_{i}\right)=\left\{\begin{array}{cc} 1, & \text{if}\;{\bar{y}}_{i}\;\text{is}\;\text{identical}\;\text{to}{\;y}_{i},\\ 0, & \text{other} \end{array}\right.$$

Precision is a metric that gauges the accuracy of a model’s predictions by comparing the number of labels correctly identified as positive against the total number of positive predictions made. A model demonstrating high precision is one whose positive predictions can be trusted to be accurate. Meanwhile, accuracy is a broader metric, indicating the proportion of all labels—both positive and negative—that are correctly identified by the model, thereby serving as a direct indicator of the model’s overall predictive performance. Coverage, on the other hand, compares the number of correct predictions to the actual number of true labels, with high coverage suggesting the model’s effectiveness in identifying a large number of the correct labels.

The term “Absolute true” refers to the frequency at which the classifier accurately predicts the label set for each sample. A sample is considered to be completely correctly classified if the predicted label set is consistent with the true label set. On the other hand, “Absolute false” represents the frequency at which the prediction results do not match the true labels. Only when a sample’s predicted label set has no overlap with the actual label set, it will be counted as an absolute false.

### 2.3 Experimental results

#### 2.3.1 Individual hierarchical models

To evaluate the feature extraction capability of the hierarchical model of AMHF‐TP, the assessment involved separate tests on the model’s three subnetworks, namely network 1, network 2, and network 3 (as depicted in Figure 1). The results of these tests are presented in Table 2. For clarity, AMHF‐TP_1 refers to the atomic feature extraction model, AMHF‐TP_2 denotes the amino acid sequence feature extraction model, and AMHF‐TP_3 represents the multi‐sequence relational feature extraction model. This nomenclature is maintained throughout the discussion.

**TABLE 2:** Results of tests on individual hierarchical models.

Dataset	Model	Precision	Coverage	Accuracy	Absolute true	Absolute false
Training	AMHF‐TP_1	0.208	0.178	0.178	0.156	0.055
	AMHF‐TP_2	**0.676**	**0.651**	**0.628**	**0.568**	**0.035**
	AMHF‐TP_3	0.508	0.458	0.458	0.420	0.040
Test	AMHF‐TP_1	0.111	0.225	0.111	0.008	0.119
	AMHF‐TP_2	**0.659**	**0.664**	**0.621**	**0.551**	**0.039**
	AMHF‐TP_3	0.450	0.482	0.423	0.358	0.055

*Note*: Bold values in the table indicate the best results obtained among all compared algorithms.

As shown in Table 2, AMHF‐TP_2 has the highest precision on the training set (0.676) and maintained high precision on the test set (0.659), indicating strong positive case prediction and generalization capabilities. It also surpassed the other models in accuracy and other metrics, highlighting the importance of sequence features in peptide identification and the effectiveness of combining CNNs with self‐attention mechanisms.

However, AMHF‐TP_1 showed significantly lower performance, suggesting potential information loss during amino acid to atom conversion. AMHF‐TP_3, focusing on inter‐sequence relationships, improved upon AMHF‐TP_1 but lagged behind AMHF‐TP_2, potentially due to disrupted sequence integrity in its hypergraph construction process.

#### 2.3.2 Feature fusion experiment of three models

This section tests the performance of different combination models for feature extraction from the three hierarchical models (network 1, network 2, and network 3 in Figure 1). The results are presented in Table 3, where AMHF‐TP_12 represents the combination of AMHF‐TP_1 and AMHF‐TP_2, AMHF‐TP_13 represents the combination of AMHF‐TP_1 and AMHF‐TP_3, and so on. AMHF‐TP_123 represents the combination of AMHF‐TP_1, AMHF‐TP_2, and AMHF‐TP_3.

**TABLE 3:** Test results of feature fusion.

Model	Dataset	Precision	Coverage	Accuracy	Absolute true	Absolute false
Training	AMHF‐TP_12	0.660	0.665	0.622	0.552	0.039
	AMHF‐TP_13	0.512	0.462	0.462	0.425	0.040
	AMHF‐TP_23	0.918	0.920	0.905	0.877	0.011
	AMHFTP_123	**0.924**	**0.919**	**0.911**	**0.891**	**0.009**
Test	AMHF‐TP_12	0.652	0.641	0.612	0.550	0.037
	AMHF‐TP_13	0.503	0.487	0.464	0.411	0.055
	AMHF‐TP_23	0.914	0.915	0.899	0.869	0.012
	AMHFTP_123	**0.915**	**0.916**	**0.900**	**0.871**	**0.012**

*Note*: Bold values in the table indicate the best results obtained among all compared algorithms.

As shown in Table 3, the AMHF‐TP_123 model, which combines all three layers, achieved the highest performance on the test set with 91.5% precision and the best results in other metrics, demonstrating high accuracy in prediction tasks. The AMHF‐TP_23 model followed closely, showing significant improvement over the single AMHF‐TP_2 model. This indicates that the information extracted by AMHF‐TP_3 plays a significant role in improving the overall performance of the model, emphasizing the positive impact of multi‐sequence relational extraction based on hypergraphs on the overall model performance. The AMHF‐TP_123 model performed slightly better than AMHF‐TP_23, suggesting a positive but minor role of atomic sequence features from AMHF‐TP_1. The AMHF‐TP_12 model showed moderate performance, while AMHF‐TP_13 was the least effective, indicating that the features from AMHF‐TP_1 might have a mixed impact on the other models.

Tables 2 and 3 show that AMHF‐TP_23 outperforms AMHF‐TP_2 and AMHF‐TP_3. AMHF‐TP_2 is a model that extracts amino acid sequence and secondary structure features using CNNs and self‐attention mechanisms. AMHF‐TP_3, on the other hand, is a model that extracts multi‐sequence relationship features. The multi‐sequence relationship features of MFTP are extracted by constructing hypergraphs and utilizing a hypergraph attention network.

AMHF‐TP_2’s CNN efficiently captures local features of amino acid sequences and their secondary structures, while the self‐attention mechanism enhances the model’s understanding of global dependencies. However, its perspective remains limited to individual sequences, despite its strong performance in single‐sequence feature extraction. AMHF‐TP_3 introduces hypergraph construction technology, which integrates the relationships among multiple sequences, establishing complex networks. This helps the model capture higher‐order similarities and differences between sequences. By utilizing a hypergraph attention network to extract multi‐sequence relationship features, this method comprehensively captures the key features of peptide sequences. This is significant for identifying peptides with similar functions or structures. The integration of features of different granularities from AMHF‐TP_2 and AMHF‐TP_3 in AMHF‐TP_23 enhances the diversity and complexity of feature representation. It helps to achieve higher accuracy in a complex biological sequence analysis by aiding in a deeper understanding of the complex interactions between MFTPs.

#### 2.3.3 Comparison with existing models

In this section, AMHF‐TP (AMHF‐TP123) is compared with existing models MPMABP [15], MLBP [17], SP‐RNN [16], PrMFTP [18], and ETFC [19] as shown in Table 4. Some of the data are derived from literature [19].

**TABLE 4:** Comparison with existing models.

Model	Precision	Coverage	Accuracy	Absolute true	Absolute false
MPMABP	0.477	0.444	0.430	0.381	0.041
MLBP	0.549	0.498	0.493	0.446	0.037
SP‐RNN	0.604	0.620	0.566	0.482	0.038
PrMFTP	0.699	0.669	0.651	0.593	0.031
ETFC	0.724	0.717	0.684	0.617	0.036
AMHF‐TP	**0.915**	**0.916**	**0.900**	**0.871**	**0.012**

*Note*: Bold values in the table indicate the best results obtained among all compared algorithms.

As shown in Table 4, the AMHF‐TP model shows outstanding performance across all evaluation metrics, with precision and coverage exceeding 0.9 and accuracy reaching an impressive 0.900. The improvements in precision, coverage, and accuracy provided by AMHF‐TP highlight its advanced predictive capabilities and broad applicability in identifying MFTP with precision.

The efficacy of AMHF‐TP can be attributed to its innovative use of hypergraph technology. This technology is employed for the extraction of multi‐sequence relationship features, which are then combined with the extraction of amino acid sequence features. The latter is achieved through a harmonious blend of CNNs and self‐attention mechanisms. This multilevel fusion of features is pivotal in capturing a broad spectrum of peptide sequence characteristics.

The hypergraph component of AMHF‐TP presents a groundbreaking approach to understand and represent the complex interconnections among multiple peptide sequences. Through the construction of hypergraphs and the application of hypergraph attention networks, AMHF‐TP excels in discerning intricate patterns and dependencies across sequences. Traditional single‐sequence approaches fall short of this task. The model’s ability to comprehend higher‐order relationships significantly bolsters its precision and generalization in predicting MFTP.

Moreover, the utilization of CNNs and self‐attention mechanisms for the extraction of amino acid sequence features guarantees a thorough and nuanced analysis of both local and global sequence characteristics. This strategy empowers AMHF‐TP to identify both established and potentially novel peptide functions through its rich, multilevel feature representation.

Furthermore, the model’s hierarchical feature extraction method processes features at various granularities, culminating in a more robust and diverse feature representation. This method enhances the model’s capacity to recognize and distinguish between peptides with different functions, thereby augmenting its predictive accuracy and generalization capabilities.

## 3 DISCUSSIONS

The AMHF‐TP model represents a significant advancement in our understanding and representation of peptide sequence complexity, thanks to its innovative multilevel feature extraction approach. By optimizing different feature dimensions at each layer, the model enhances its performance and broadens its applicability across various domains within bioinformatics and drug design. The model’s efficient recognition of MFTP is set to accelerate drug development processes and contribute to the advancement of personalized medicine.

However, the integration of advanced methodologies introduces challenges, including the need for comprehensive, high‐quality datasets for model training and potential increases in computational complexity. The model’s performance and its ability to generalize across diverse peptide types can be significantly influenced by the quality and breadth of the training data. Moreover, the computational demand of processing through both hypergraphs and deep learning architectures necessitates careful consideration, particularly for large‐scale applications.

Future research will explore enhancing hypergraph construction and improving atomic‐level feature extraction and focus on refining the model’s efficiency and scalability, exploring ways to mitigate computational demands while preserving, or even enhancing, performance. Efforts will also be made to broaden the model’s adaptability to varied and less common peptide sequences, possibly through innovative training strategies or the incorporation of more sophisticated machine learning techniques.

## 4 CONCLUSIONS

The proposed AMHF‐TP is a multifunctional therapeutic peptide recognition method that leverages attention mechanisms and multi‐granularity hierarchical features. It is composed of three distinct subnetworks: an atomic sequence feature extraction network, an amino acid sequence feature extraction network, and a multi‐sequence relational feature extraction network. Each network addresses different aspects of peptides, namely atomic composition, amino acid sequences, and relational features, and presents unique strengths and limitations.

The atomic sequence network leverages transfer learning for robust feature extraction from minimal data, but it may not fully capture complex amino acid interactions. The amino acid sequence network, integrating CNNs and self‐attention mechanisms, excels at identifying sequence patterns but may miss intricate relational structures. The multi‐sequence relational network maps complex peptide relationships using a hypergraph attention network, but it might overlook the compositional information within peptide sequences.

The outputs of these subnetworks are integrated through a fully connected layer, enabling the model to learn from the diverse feature sets. This fusion approach harnesses the full potential of the multi‐granularity features, significantly enhancing the model’s ability to recognize MFTP. Experimental evaluations underscore the importance of combining features from different levels of granularity for an effective peptide recognition. The results affirm the effectiveness and robustness of AMHF‐TP in identifying multifunctional peptides as it outperforms existing models across various metrics.

## 5 MATERIALS AND METHODS

### 5.1 Related works

#### 5.1.1 Self‐attention mechanism and text convolution

Self‐attention, a crucial technique in deep learning widely used in natural language processing (NLP) and computer vision, dynamically assigns attention weights while processing sequence data, capturing relationships and dependencies between elements. The process involves linearly mapping elements into *Q*, *K*, and *V* vectors, calculating similarity scores between *Q* and *K*, normalizing weights using softmax, and generating attention representations. MHSA extends this by learning multiple sets of *Q*, *K*, and *V* weights, thereby enhancing information representation [20]. However, it faces challenges such as difficulty in capturing local dependencies and high computational complexity. Combining CNNs with self‐attention can improve sensitivity to local features and capture diverse features (global dependencies and local features), enhancing expressiveness and generalization. In this study, text convolution (TextCNN) [21] is incorporated for feature extraction from amino acid sequences and secondary structures, leveraging local convolutional layers to capture varying lengths of local dependencies in texts. This combination in peptide research enables better understanding of contextual features and hidden relationships in amino acid sequences.

#### 5.1.2 Bidirectional Encoder Representations from Transformers

BERT (Bidirectional Encoder Representations from Transformers) is a groundbreaking advancement in NLP [22]. The main innovation of BERT lies in its bidirectional pretraining model, which utilizes the transformer architecture to take into account both the left and right context of words. This approach enables a more comprehensive understanding of the relationships and contexts of words. BERT’s operational workflow consists of two main phases: pretraining and fine‐tuning [23]. During the pretraining phase, the model is trained on a large text corpus, such as Wikipedia, to acquire a deep understanding of language representations. A crucial task in this phase is the masked language model task. Fine‐tuning, on the other hand, customizes the model for specific tasks by replacing the final layers with task‐specific classification layers [24]. In this study, a pretrained BERT model is fine‐tuned using peptide sequences converted into atomic sequences. This process allows for the extraction of contextual features that are specific to atomic sequences. By leveraging prior knowledge, the model is able to identify potential relationships in peptide sequences [25].

#### 5.1.3 Hypergraph

Hypergraphs [26] are generalizations of graphs that can efficiently capture many‐to‐many relationships. In contrast to graphs, where edges link pairs of nodes, hypergraphs allow edges (called hyperedges) to connect arbitrary sets of nodes. This makes hypergraphs more suitable for modeling complex data and relationships. The key component of a hypergraph is its hyperedges, which can represent higher‐order relations or interactions among multiple nodes. The nodes in a hypergraph denote entities or elements in the data and can belong to one or more hyperedges, forming a complex network structure. Hypergraphs have been applied to various domains in bioinformatics, such as protein–protein interaction networks, gene regulatory networks, and metabolic networks. Hypergraphs enable a more comprehensive analysis of the correlations among different types of data, the identification of key nodes and pathways in protein and metabolic networks, and the prediction of regulatory mechanisms in biological processes [27, 28, 29, 30, 31].

In this study, to learn the similarity of sequences for sequence classification, it is necessary to define the relationships between sequences and the relationships between subsequences within each sequence. To achieve this, a hypergraph can be constructed to capture the higher‐order similarity of sequences. In hypergraph, subsequences are represented as nodes. Each sequence containing a group of subsequences constitutes a hyperedge. Each hyperedge can connect to other hyperedges through some shared nodes as subsequences. By constructing the hypergraph in this manner, higher‐level connections between sequences and subsequences can be defined, aiding in the capture of the complex similarity between sequences [32, 33].

### 5.2 Methodology outline

This study presents a novel approach, called AMHF‐TP, for identifying peptides with multifunctional therapeutic properties. The approach utilizes an attention mechanism and multi‐granularity hierarchical feature learning to enhance peptide classification performance.

As shown in Figure 1, the proposed AMHF‐TP comprises three components, each operating on different levels of representation for multifunctional peptide sequences.

#### 5.2.1 Atomic‐level feature extraction (network 1 of Figure 1)

This component employs transfer learning to extract atomic‐level features from the amino acid atomic composition. It utilizes the RDKit tool to compute molecular descriptors for each amino acid and applies a specific tokenizer model to convert them into tokens. These tokens are input into the Sci‐BERT [34] pretrained model, a BERT model fine‐tuned on scientific literature, to obtain rich and contextualized features that capture the fine‐grained mechanisms of action within sequences.

#### 5.2.2 Sequence‐level feature extraction (network 2 of Figure 1)

This component uses CNNs and a self‐attention mechanism to extract sequence‐level features from the amino acid sequences and their secondary structures. It first encodes the raw sequence features and the corresponding secondary structure features using an embedding layer, then passes them separately through a MHSA mechanism and a TextCNN model. The self‐attention mechanism enables the model to learn the dependencies and interactions between different parts of the sequences, while the TextCNN model extracts local and global features using convolutional filters of varying sizes. The output of this component is a comprehensive representation of the amino acid sequences.

#### 5.2.3 Relational feature extraction (network 3 of Figure 1)

This component uses a hypergraph to capture high‐order similarities between sequences and subsequences. It aims to model the complex and nonlinear relationships between sequences that share common subsequences or motifs, which are crucial for multifunctionality. To construct the hypergraph, all peptide sequences are first split into subsequences of a fixed length, and each subsequence is assigned as a node in the hypergraph. The edges in the hypergraph are defined as sets of nodes that belong to the same original sequence, and the weights of the edges are computed based on the frequency and diversity of the subsequences. A hypergraph attention feature extraction model, an extension of graph attention networks, is then applied to learn relational features of multiple sequences from the hypergraph structure.

The final stage of the AMHF‐TP is to fuse the features from the previous layers and perform the final classification. The method extracts three types of features: atomic features that capture the physical and chemical properties of each amino acid, sequence features that encode the order and composition of amino acids in a peptide, and relational features that represent the similarities and differences among multiple peptide sequences. These features are extracted using three corresponding networks: the atomic feature network (network 1), the sequence feature network (network 2), and the hypergraph network (network 3). A feature fusion network is used to integrate these features and obtain a comprehensive representation of each peptide by combining them using a weighted sum. The weights are learned by the network based on the importance of each feature type for the classification task. The output of the feature fusion network is then fed into a sigmoid layer that predicts the probability of each peptide belonging to one of the multifunctional classes.

### 5.3 Atomic feature extraction based on transfer learning

This part of the AMHF‐TP involves the extraction of the finest‐grained features (atomic level) in the multi‐granularity hierarchical feature extraction process. Unlike previous studies that only focused on amino acid sequences, this part explores the potential relationships between the atomic components of functional peptides. To achieve this, we use transfer learning to fine‐tune a BERT pretrained model Sci‐BERT on the atomic sequences of functional peptides and extract their features. The aim is to discover hidden patterns that may exist at the lowest level of granularity.

**FIGURE 2:**
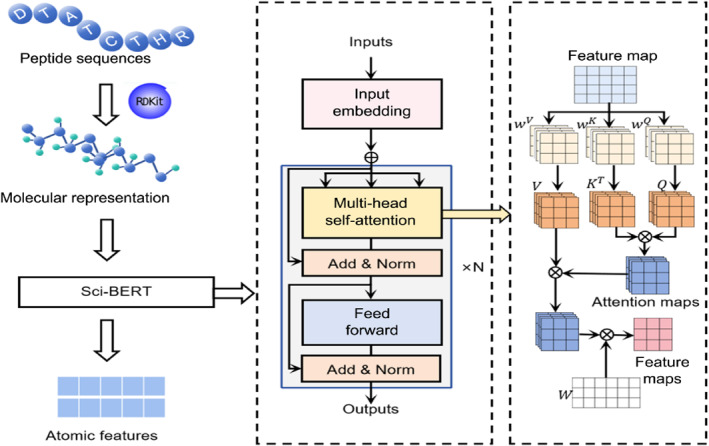
Atomic feature extraction based on transfer learning. First, the original amino acid sequence data need to be calculated using the RDKit tool to obtain the corresponding SMILES molecular formula, which represents the amino acid atomic composition sequence. Then, the transformed atomic sequence data are fed into the Sci‐BERT pretrained model for feature extraction. In the Sci‐BERT architecture, the atomic sequence data are taken as input and passed through multiple layers of network structures consisting of multi‐head self‐attention and feed forward. Finally, atomic features are obtained.

As shown in Figure 2, atomic feature extraction based on transfer learning includes two steps.

#### 5.3.1 Amino acid molecular representation calculation based on RDKit tool

RDKit is an open‐source toolkit widely used in cheminformatics and molecular modeling. It offers a rich set of functionalities for processing, analyzing, and visualizing chemical molecules and related data. RDKit has various powerful features, including molecular representation, chemical computation, molecular visualization, and drug discovery. In terms of molecular representation, RDKit supports multiple methods, such as SMILES, Mol objects, and SMARTS, making the standardization and storage of molecules simple and conducive to further analysis and processing.

**FIGURE 3:**
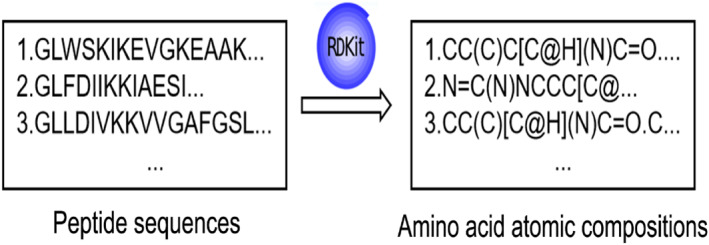
Amino acid molecular representation. The peptide sequence data are converted into corresponding atomic sequence data using the RDKit tool through computation.

As shown in Figure 3, the RDKit is mainly used for processing amino acid data. The Chem module is a crucial part of RDKit for handling and manipulating chemical molecular representations, conversions, and calculations. It has functionalities for creating, reading, and manipulating SMILES molecular formulas and Mol objects. First, the module’s methods are used to convert amino acid sequence features into molecular objects. Then, a molecular traversal is performed starting from the initial atom. This method moves along the molecular topological structure, traversing atoms and bonds. The starting atom can be any atom, usually a carbon atom in the molecule. During this process, it adds information about atoms and bonds to the generated SMILES string one by one and performs a series of special treatments. For example, for molecules containing aromatic rings, the method recognizes aromaticity and adjusts the SMILES representation accordingly to ensure the correct display of aromaticity. As shown in Figure 3, using the RDKit tool, the amino acid sequence can be converted into the atomic sequence.

#### 5.3.2 Atomic‐level feature extraction based on BERT pretrained model

To enhance the representation of atomic‐level features, we employ Sci‐BERT as the backbone model for our proposed AMHF‐TP. Sci‐BERT is a variant of BERT, one of the most powerful NLP models that can learn rich contextual embeddings from large‐scale unlabeled text data. BERT is based on the transformer architecture, which consists of multiple layers of self‐attention mechanisms that enable the model to attend to different parts of the input sequence. By doing so, BERT can capture both syntactic and semantic information from text data, and it can be fine‐tuned for various downstream tasks.

However, BERT is not optimized for scientific texts, which have different linguistic patterns and domain‐specific vocabularies than general texts. Therefore, Sci‐BERT was proposed to address this gap by pretraining BERT on a large corpus of scientific papers from diverse domains. Sci‐BERT was trained on 1.14 million random samples of scientific papers, with 18% from computer science and 82% from biomedical fields. The corpus covers not only abstracts but also full texts of the papers, resulting in a dataset size comparable to that of BERT. By pretraining on such a corpus, Sci‐BERT can learn the semantic and syntactic features of scientific language, and it can also acquire a wealth of biomedical domain knowledge. Moreover, Sci‐BERT can better handle SMILES molecular formulas, which are commonly used to represent chemical structures in text data.

Therefore, we choose Sci‐BERT as the model for extracting atomic‐level features from SMILES sequences as it can effectively encode the structural and functional information of molecules.

As shown in Figure 2, the atomic sequence data are passed into the Sci‐BERT pretrained model for feature extraction. Within the Sci‐BERT architecture, the atomic sequence data are taken as inputs and passed through an embedding encoding before being fed into the N‐layer network structure of the MHSA. Within the MHSA, the embedded feature maps are processed using weight matrices to compute corresponding *Q*, *K*, and *V* matrices, followed by self‐attention computation to obtain feature maps. Additionally, the data undergoes further computations through other network layers, such as residual connections, normalization structures (Add norm), and fully connected structures (Feed forward).

To adapt Sci‐BERT for predicting molecular properties based on SMILES molecular formulas, we added two fully connected layers to extract features from the SMILES representation. Additionally, we applied a “Dropout” layer to prevent overfitting and used BertAdam as an optimizer, which includes weight decay and preheating. The batch size was set to 128, the learning rate to 5e–6, and the number of iterations to 30.

### 5.4 Amino acid sequence feature extraction based on CNN and self‐attention mechanism

In the AMHF‐TP model, amino acid sequence feature extraction is based on the CNN combined with a self‐attention mechanism. Unlike previous approaches that only utilized the primary sequences as input features, our method also incorporates the secondary structure information of the amino acid sequences, which is computed using the ChouFasman algorithm [33]. The feature extraction process consists of two parallel pathways: one for extracting the intrinsic features of the amino acid sequences and another for extracting the structural features of the amino acid sequences.

**FIGURE 4:**
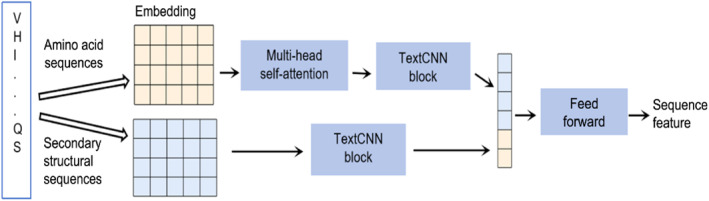
Amino acid sequence feature extraction. First, the obtained amino acid sequences and the secondary structure sequences calculated using the ChouFasman algorithm are separately embedded; then, different channels of feature extraction are performed. In the process of amino acid sequence feature extraction, a combination of the multi‐head self‐attention mechanism and TextCNN is mainly adopted to extract a sequence feature. In the structure feature extraction process, only TextCNN is used to extract the secondary structure features. Finally, feed forward is used to further integrate the features, thereby obtaining the final amino acid sequence features.

Figure 4 illustrates the architecture of the feature extraction model based on CNN and the self‐attention mechanism. The model takes two types of input features, sequence features and structure features, and applies different feature extraction layers. The details of these two feature extraction pathways are described as follows.

#### 5.4.1 Sequence feature extraction

Sequence feature extraction is a crucial step for predicting the function of peptides. It involves two primary steps.

##### Transforming peptide sequences into a feature matrix using embedding techniques

To encode the peptide sequences, we first assign a numerical value to each of the 20 amino acids (A, C, D, E, F, G, H, I, K, L, M, N, P, Q, R, S, T, V, W, and Y) based on their single‐character representation. For example, A is encoded as 1, C as 2, and so on. Since the peptide sequences in the benchmark dataset vary in length from 5 to 50 amino acids, we pad the shorter sequences with “0” and truncate the longer ones to ensure a consistent input length of 50. Next, a semantic embedding method for amino acid sequences is applied to transform the numerical encoding matrix into a semantic embedding matrix (*n* = 50, *d* = 192), which captures the biological properties and functional roles of each amino acid. Finally, the position information for each amino acid is computed using a transformer‐based position‐embedding algorithm resulting in a position embedding matrix *P* with the same dimensions as the semantic embedding matrix.

##### Importing the sequence feature matrix into a feature extraction model for feature extraction

The feature extraction model takes the semantic embedding matrix *X* and the position embedding matrix *P* as inputs and applies a combination of MHSA and TextCNN to extract features from the sequence feature matrix. MHSA is a transformer‐based decoder architecture that captures remote dependencies between amino acids, which is the same structure as MHSA in Sci‐BERT. TextCNN is a multiscale CNN that uses different sizes of convolutional filters to obtain local features of varying lengths from the embedded matrix representations. The convolutional filter sizes are chosen to match the minimum length of peptide sequences in the benchmark dataset, which is five amino acids. Therefore, the filter sizes are 2, 3, 4, and 5. After the convolution operation, a max‐pooling layer is applied to reduce the dimensionality of the feature vectors. The final output is a feature representation that incorporates the global features of the peptide sequences.

#### 5.4.2 Structural feature extraction

Figure 4 shows that structural feature extraction involves two main steps.

##### Obtaining the secondary structure features

The ChouFasman algorithm [35] is employed to predict secondary structures from amino acid sequences. It assigns each amino acid to a basic structural element, such as α‐helix, β‐sheet, or random coil, based on the probabilities derived from a large dataset of known protein structures. The algorithm consists of three steps:

1. *Obtaining the ChouFasman table*. The ChouFasman table contains the probability parameters for each amino acid to form a certain secondary structure within a window of adjacent amino acids.
2. *Performing sliding window analysis*. This step calculates the potential structures for each position in the protein sequence by sliding a window along the sequence and using the probability parameters from the table.
3. *Determining structural element regions*. This step identifies the regions where the potential structures are consistent and significant.

By applying this algorithm, one can generate secondary structure sequence features from amino acid sequence data. In this study, we use four categories of secondary structures: α‐helix, β‐sheet, β‐turn, and random coil, encoded as 0–3, respectively.

##### Passing the structural feature sequence into the TextCNN feature extraction model for feature extraction

The same embedding calculation method and TextCNN structure used in the sequence feature extraction process are applied since the structural feature data has the same dimensions as the sequence feature data. The only difference in the structural feature extraction process is the reduction of the MHSA block in the feature extraction process. The prediction of protein secondary structures is influenced by many complex factors. The ChouFasman algorithm is a relatively simple method with certain limitations and limited accuracy. Therefore, it is advisable to perform a preliminary extraction of the information provided by the algorithm. This approach provides the model with necessary information while avoiding the influence of any interfering data that might affect the model’s extraction process.

### 5.5 Multi‐sequence relation feature extraction based on hypergraph attention network

To capture the relationships between multiple peptide sequences, AMHF‐TP adopts a hypergraph approach that differs from the previous two models that only extracted features at the atomic and amino acid levels. This model can leverage a hypergraph attention network to learn more sophisticated and informative features from the peptide data.

**FIGURE 5:**
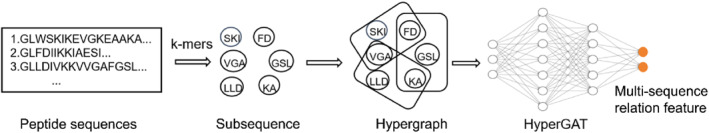
Multi‐sequence relation sequence feature extraction. First, the peptide sequences are divided into subsequences using k‐mers. Then, these divided subsequences are used as nodes to construct a hypergraph. Subsequently, the constructed hypergraph is used as an input, and a two‐stage feature updating process of the HyperGAT is employed for feature learning. These stages include node feature updating and hyperedge feature updating. Finally, multiple sequence relationship features are obtained. HyperGAT, hypergraph attention network.

Figure 5 illustrates the multi‐sequence relational feature extraction process based on the hypergraph attention network, which consists of two parts: (1) subsequence division using k‐mers; (2) constructing a hypergraph and using the hypergraph attention feature extraction model to extract features. The details of each part are as follows.

#### 5.5.1 Subsequence division using k‐mers

**FIGURE 6:**
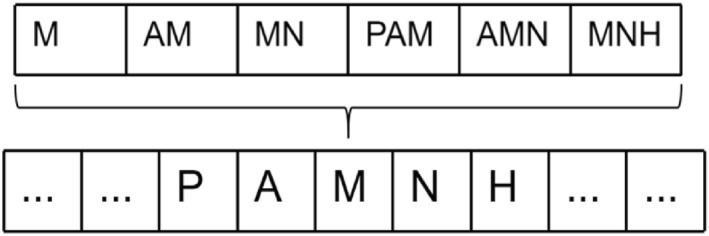
Subsequence division using k‐mers. The k‐mers method is a very useful sequence analysis technique. Specifically, starting from the first character, then moving only one character to obtain the next subsequence, and so on. In the sequence “PAMNH,” using “M” as an example, the 1‐mer would be “M,” 2‐mers: “AM, MN,” and 3‐mers: “PAM, AMN, MNH.” In this work, the 5‐mers tokenization method is mainly used.

The k‐mers method is a common technique for processing sequence data (such as DNA, RNA, or protein sequences). It involves dividing the sequence into continuous, overlapping subsequence segments, which typically contain k consecutive nucleotides or amino acids. These subsequence segments are known as k‐mers. The k‐mers method is a very useful sequence analysis technique, applicable to various fields, especially in bioinformatics for DNA and protein sequence analysis. It helps extract important sequence features and reveal similarities and structures between sequences. Specifically, as shown in Figure 6, starting from the first character, then moving only one character to obtain the next subsequence, and so on. For example, in the sequence “PAMNH,” using “M” as an example, the 1‐mer would be “M,” 2‐mers: “AM, MN,” and 3‐mers: “PAM, AMN, MNH.” In this work, the 5‐mers tokenization method is mainly used.

#### 5.5.2 Constructing a hypergraph and using the hypergraph attention feature extraction model to extract features

To learn sequence similarities and extract more latent features of peptide sequences, it is necessary to define the connections between sequences and the connections between subsequences within a sequence. For this, the hypergraph method can be used to capture higher‐level sequence similarities.

**FIGURE 7:**
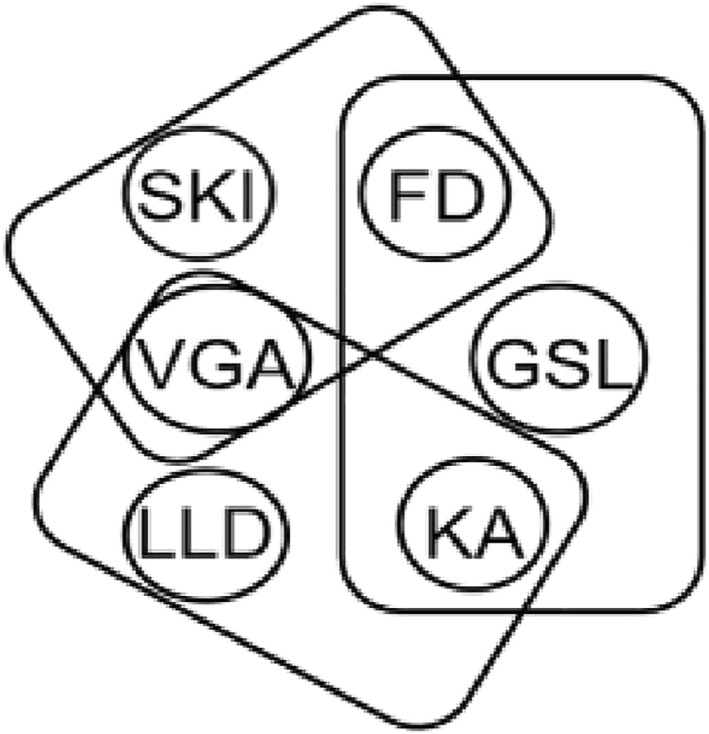
Constructing the hypergraph. The graph consists of three peptide sequences: SKIVGAFD, VGALLDKA, and FDGSLKA. Each peptide sequence contains different subsequences, and the relationships between these sequences are constructed based on the subsequences they share.

As shown in Figure 7, the subsequences divided by the k‐mer algorithm are represented as nodes in the hypergraph. Each sequence containing respective subsequences becomes an edge in the hypergraph. For example, there are three sequences in the figure, and their relationships are established through shared subsequences. In the hypergraph of this study’s model, which includes all peptide sequence data, each hyperedge represents a peptide sequence sample. These hyperedges can connect through shared nodes, representing the similarity between sequences. Through this method of constructing a hypergraph, we can capture higher‐level connections between sequences, aiding in understanding the complex similarities among multiple sequences.

The hypergraph attention network (HyperGAT) is an attention mechanism for processing graph data, extending the traditional graph attention mechanisms to enable multi‐to‐multi interactions between nodes in a network. It consists of two feature‐updating processes: updating node feature representations by learning the weights of hyperedges and updating the weights of hyperedges by learning node feature representations.

Hypergraph *G* = (*V*, *E*), *G* consists of a set of nodes *V* and a set of hyperedges *E*, where each hyperedge *e* ∈ *E* can associate with any number of nodes. The hypergraph can be represented using an association matrix *H*, where *H* ∈ {0, 1}^|*E*|×|*V*|^. If node *v*_*i*_ belongs to hyperedge *e*_*j*_, then *H*_*ij*_ = 1, otherwise *H*_*ij*_ = 0.

In the node feature update stage, we focus on how to update each node’s feature $h_{v_{i}}$. The new feature $h_{v_{i}}^{\prime }$ of the node is calculated using the following process:

(3) $$h_{v_{i}}^{\prime }=\sigma \left(\sum_{e_{j}\in E\left(v_{i}\right)}\alpha_{ij}W_{e}h_{e_{j}}\right)$$

Here, *E*(*v*_*i*_) is the set of all hyperedges that contain node *v*_*j*_, $h_{e_{j}}$ is the feature vector of hyperedge *e*_*j*_, *W*_*v*_ and *W*_*e*_ are the linear transformation matrices for node features and hyperedge features, respectively. The attention coefficient *α*_*ij*_ represents the influence of hyperedge *e*_*j*_ on node *v*_*i*_, which can be expressed as follows:

(4) $$\alpha_{ij}=\text{softmax}\left(\text{LeakyRelU}\left(W_{v}h_{v_{i}}⊙W_{e}h_{e_{j}}\right)\right)$$

The update process of hyperedge features focuses on how to use the features of the nodes it connects to update the feature $h_{e_{j}}$ of each hyperedge *e*_*j*_. The updated hyperedge feature $h_{e_{j}}^{\prime }$ can be obtained through the following method:

(5) $$h_{e_{j}}^{\prime }=\sigma \left(\sum_{v_{j}\in e_{i}}\beta_{ji}W_{v}^{\prime }h_{v_{j}}\right)$$

Here, *v*_*j*_ is the node connected to hyperedge *e*_*i*_, $W_{e}^{\prime }$ and $W_{v}^{\prime }$ are another set of linear transformation matrices for hyperedge features and node features, respectively. The attention coefficient *β*_*ji*_ represents the contribution of node *v*_*j*_ to the feature update of hyperedge *e*_*i*_, and it can be calculated as follows:

(6) $$\beta_{ji}=\text{softmax}\left(\text{LeakyReLU}\left(W_{v}^{\prime }h_{v_{i}}⊙W_{e}^{\prime }h_{e_{i}}\right)\right)$$

In these two processes, the softmax function is applied either on all nodes *v*_*j*_ within hyperedge *e*_*i*_ or on all hyperedges *e*_*i*_ associated with node *v*_*j*_. This ensures that the attention coefficients are normalized within their respective neighborhoods.

### 5.6 Multi‐granularity hierarchical feature fusion extraction

To obtain the final sequence probability scores for each therapeutic peptide class, we fused and extracted the multi‐granularity hierarchical features that were derived from the previous three networks. The fusion process involved concatenating three types of features: (1) the atomic‐level features that were learned by pretrained Sci‐BERT in network 1, (2) the amino acid sequence features that were learned by CNNs and self‐attention mechanisms in network 2, and (3) the multi‐sequence relational features that were captured by hypergraph attentional network in network 3. After concatenating these features, we fed them to a feature extractor composed of multiple fully connected layers to extract the fused features. Finally, we used a sigmoid activation function to generate the sequence probability scores for each of the 21 classes of therapeutic peptides. We assigned the predicted label for each class based on a threshold value of 0.5.

## AUTHOR CONTRIBUTIONS

**Shouheng Tuo**: Methodology; project administration; supervision; writing – review & editing. **YanLing Zhu**: Methodology. **Jiangkun Lin**: Validation. **Jiewei Jiang**: Funding acquisition.

## CONFLICT OF INTEREST STATEMENT

The authors Shouheng Tuo, YanLing Zhu, JiangKun Lin, and Jiewei Jiang declare that they have no conflict of interest or financial conflicts to disclose.

## ETHICS STATEMENT

This article does not contain any studies with human or animal materials performed by any of the authors.

## Data Availability

AMHF‐TP is available on the Github website (shouhengtuo/AMHF‐TP). The datasets generated and/or analyzed during the current study are available from the corresponding author upon reasonable request. We have adhered to all relevant data sharing policies and will ensure that the data is accessible to researchers in a timely and responsible manner, subject to any necessary restrictions imposed by privacy, confidentiality, or intellectual property considerations.
